# Predictors of sleepiness in a large-scale epidemiology study ESSE-RF

**DOI:** 10.3389/fneur.2024.1431821

**Published:** 2024-09-04

**Authors:** Mikhail Bochkarev, Lyudmila Korostovtseva, Oxana Rotar, Elena Verbitskaya, Yurii Sviryaev, Yulia Zhernakova, Svetlana Shalnova, Alexandra Konradi, Irina Chazova, Sergey Boytsov, Evgeny Shlyakhto

**Affiliations:** ^1^Almazov National Medical Research Centre, Saint Petersburg, Russia; ^2^Pavlov First Saint Petersburg State Medical University, Saint Petersburg, Russia; ^3^National Medical Research Center for Cardiology, Moscow, Russia; ^4^National Medical Research Centre for Therapy and Preventive Medicine, Moscow, Russia

**Keywords:** sleep, sleepiness, epidemiology, sleep disorders, insomnia

## Abstract

**Introduction:**

To identify predictors of excessive daytime sleepiness we analyzed data from the ‘Epidemiology of cardiovascular diseases in regions of Russia (ESSE-RF)’ study.

**Methods:**

Data from participants of the cohort study ESSE-RF (2012–2013), aged 25–64 years, from 13 regions of Russia were analyzed (2012–2013). The participants were interviewed regarding their sleep complaints, including difficulties with initiating and maintaining sleep, sleepiness, and use of sleeping pills. Sleepiness was considered significant if it occurred at least three times a week. The examination encompassed social, demographic, and anthropometric measures, lifestyle factors, self-reported diseases, and laboratory parameters. The final analysis included 13,255 respondents.

**Results:**

Frequent (≥3 times/week) sleepiness was reported by 5,8%, and occasional sleepiness (1–2 times/week) by 10.8% of respondents. Multivariate regression analysis identified significant predictors of frequent sleepiness. Sleep complaints (insomnia, sleep apnea, snoring) and frequent use of sleep medication were prominent factors. Additionally, age, female gender, higher education, and retirement status were associated with sleepiness. Beyond demographics and sleep, the analysis revealed predictors: abnormal anxiety levels, low high-density lipoprotein, high salt intake and following medical conditions: arrhythmia, hypertension, myocardial infarction, other heart diseases, and renal disease.

**Conclusion:**

This study identified a significant prevalence of EDS in Russians, aligning with global trends. However, findings suggest potential regional variations. Analysis revealed a complex interplay of factors contributing to EDS, highlighting the importance of individualized treatment approaches for improved sleep health.

## Introduction

Sleepiness is a clinically important symptom that characterizes the quality of sleep and is an essential feature of most sleep disorders. It is associated with a variety of factors and conditions. In a recent review of hypersomnolence, excessive daytime sleepiness (EDS) is defined as the complaint of an inability to stay awake during the normal waking hours of the day (1). EDS is associated with negative consequences on an individual’s quality of life, work performance and overall health. In order to develop effective interventions and improve the well-being of those affected, it is essential to identify risk factors and understand the prevalence of EDS (2).

Excessive daytime sleepiness (EDS) is a common complaint affecting a significant proportion of the general population. Prevalence rates range from 2.2 to 33% in different studies (3–5). This variation is due to differences in the populations studied and how EDS is defined and assessed.

Several risk factors have been identified, such as age, gender, lifestyle factors, social and medical conditions, use of medication, psychological stress and sleep-related factors (1–5). However, the relationships between these factors and the importance of their contribution to the development of EDS are complex and not yet fully understood.

Diagnostic tools, including surveys or questionnaires, or clinical approaches are used to confirm EDS. Sleepiness can be difficult to quantify because the subjective experience varies from person to person. The Epworth Sleepiness Scale is a standardized measure of subjective sleepiness that is not diagnostic but may be useful in clinical practice for quantification of sleepiness severity (6). Other recent questionnaires, such as the Barcelona Sleepiness Index (7), have shown greater accuracy in sleep-disordered breathing groups compared with objective measurement using the multiple sleep latency test.

There is great practical and scientific interest in epidemiological studies of excessive sleepiness for the identification of risk groups in the population for subsequent targeted diagnosis, treatment and prevention of psychiatric and neurological pathology. One of the first Russian multi-site epidemiological studies to investigate sleep parameters was the ESSE-RF (Epidemiological Survey of cardiovascular diSEases in different regions of the Russian Federation). This paper aims to comprehensively review the prevalence and predictors of EDS among ESSE-RF respondents.

## Methods

The study sample included participants from the population-based cohort of the Epidemiology of Cardiovascular Risk Factors and Diseases in Regions of the Russian Federation Study (ESSE-RF), conducted in 2012–2013 and covering 13 regions of the Russian Federation (8). The protocol was approved by the local ethics committee (Almazov National Medical Research Center). Approval no. 193 dated 8 October 2012. Written informed consent was obtained from all participants prior to their participation in the study. Data were collected from 13 regions of Russia with different geographical, climatic, and socioeconomic characteristics: Republic of North Ossetia-Alania (North Caucasus), Volgograd (South), Vologda (North-West), Voronezh (Central), Ivanovo (Central), Kemerovo (West Siberia), Krasnoyarsk (East Siberia), Orenburg (Volga region), Vladivostok (Far East), St Petersburg (North-West), Tomsk (West Siberia), Yekaterinburg (West Siberia), and Tyumen (Urals). Our study analyzed data from 22,258 participants who underwent the ESSE-RF survey, of which 8,541 were men (38.4%). Out of all the included subjects, 1899 (8.5%) respondents were excluded due to missing data. This included 887 respondents with missing lab test data, 542 with missing sleep module data, and 289 with inconsistent data. Additionally, 112, 16, and 53 respondents did not report their height, weight, and waist/hip ratio, respectively.

The survey collected data on various socio-demographic characteristics such as age (age groups 25–34, 35–44, 45–54, 55–64 years), gender, education, marital status, housing type, and employment status. Additionally, it included information on physical activity, diet, smoking and alcohol consumption, sleep issues, and major diseases diagnosed by a medical professional. The structured interview conducted in person by trained medical staff yielded a list of 18 major diseases, including asthma, chronic bronchitis, arthrosis, rheumatoid arthritis, hypertension, coronary heart disease, myocardial infarction, other heart diseases, arrhythmia, diabetes mellitus, ulcer, parkinsonism, stroke, organ transplant, cancer, renal disease, thyroid disease, liver, gallbladder and gastrointestinal tract diseases. The nutrition questionnaire contained questions regarding meal frequency, salt intake, and fruit and vegetable consumption. High salt intake was defined as adding salt to cooked food or eating pickles more than once a day. High fat intake was defined as favoring saturated (animal) fats in cooking and bread. Eating these foods less than once a day was defined as low fruit and vegetable intake. Alcohol abuse was defined as consumption of ≥168 g ethanol per week for men and ≥ 84 g ethanol per week for women. Low physical activity was defined as reporting less than 150 min of moderate or less than 75 min of vigorous aerobic physical activity, such as brisk walking or other equivalent activity, per week (9). The Hospital Anxiety and Depression Scale (10) was used to assess anxiety and depression. Cut-offs for increased level of anxiety and depression were eight points and higher for borderline, and 10 and higher for abnormal level.

Questions about sleep in the month prior to the survey included sleep duration (‘What was your daily sleep duration? Please include naps’), difficulty initiating sleep (‘How often have you had difficulty falling asleep within 30 min of going to bed’), and difficulty maintaining sleep (‘How often have you had difficulty going back to sleep after waking up in the middle of the night or early in the morning’). Excessive daytime sleepiness was assessed by the question ‘How often have you had difficulty staying awake when the situation required it (at work, etc.)?’ Sleeping pills use was also recorded with response options of never, less than once a week, 1–2 times a week and 3 or more times a week. The specific type of medication was not reported. Insomnia symptoms and sleepiness were defined as significant occurring at least three times a week. The analysis of sleep-disordered breathing involved the use of two questions: ‘Do you snore?’ and ‘Do you experience sleep apnea?’ Respondents were asked to answer with ‘yes’, ‘no’, or ‘do not know’. Those who answered ‘do not know’ to either question were excluded from the analysis. This resulted in the exclusion of 14% (n = 2,879) of respondents who were unsure about snoring and 24% (n = 4,197) who were unsure about sleep apnea. The final analysis included a total of 13,282 respondents.

During the interview, medical staff measured the participant’s weight and height. The body mass index (BMI) was then calculated using the Quetelet equation (weight (kg)/height^2^ (m^2^)). Height was assessed with an accuracy of up to 0.5 cm while standing, using a stadiometer (Medical RP, TVES, Russia). Weight was measured with an accuracy of up to 100 g using medical scales (HEM-150 MASSA-K, Russia). The waist circumference (WC) was measured using an inelastic 150-cm tape at the midpoint between the inferior edge of the costal border and the iliac crest in the mid-axillary line, and the hip circumference (HC) was measured at the maximum posterior protrusion of the gluteus muscles. The WHR was calculated by dividing WC (cm) by HC (cm). Hypertension was diagnosed if the office systolic blood pressure (SBP) was 140 mm Hg or higher and/or the diastolic blood pressure (DBP) was 90 mm Hg or higher, or if the patient was on antihypertensive therapy at the time of the interview.

Blood samples were collected according to standardized protocols after a minimum of 12 h of fasting on the day of the survey, prior to the interview and anthropometric measurements. The blood samples were then centrifuged at a low speed of 900 g for 20 min at +4°C. The serum was promptly frozen and stored at a temperature below −20°C until it was transferred by specialized logistics services to the laboratory at the responsible participating center. The following parameters were assessed: glucose, creatinine, and uric acid (Abbot Architect c8000, United States). The glomerular filtration rate (GFR) was estimated using the CKD-EPI formula. Total cholesterol, triglycerides, low-density lipoprotein and high-density lipoprotein were measured. The study design was previously described in detail (8).

### Statistical analysis

Statistical analyses were conducted using IBM SPSS Statistics v.16 (United States). Each parameter was assessed for normal distribution using the Kolmogorov–Smirnov test. The mean and standard deviation were presented if the parameter was normally distributed. If not, the parameter was presented as median and percentile (25%; 75%). Comparative analysis of the categorical variables was conducted using Fisher’s exact test. Homogeneity of variance was assessed using Levene’s test. Multiple comparisons were conducted using ANOVA with Welch statistics for unequal samples. Predictors of sleepiness were determined using ordinal logistic regression analysis, with sleepiness frequency included as a dependent ordinal variable. The parallel regression assumption was not met by the ordinal model [*χ*^2^(110) = 652,028, *p* < 0.001]. Therefore, multivariate regression was used, with never excessive sleepiness as the reference category. The analysis included independent variables such as 18 diseases, risk factors for sleepiness (smoking, alcohol abuse, physical inactivity, and dyslipidemia), dietary habits (high sugar, high fat, and high salt intake, low intake of fruits and vegetables), and social and demographic factors (gender, housing type, marital status, education, and employment). Covariates included continuous variables such as age, BMI, sleep duration, fasting glucose, uric acid, creatinine, office SBP and DBP, and heart rate. To avoid redundancy, closely correlated factors were not included in the same analysis. Variance inflation factor was less than 5 for all variables. GFR was excluded from analyses due to variance inflation factor was 4.15 and minimum tolerance was 0.24. Odds ratios (ORs) and 95% confidence intervals (95% CI) were estimated for each sleepiness frequency association with each factor and medical disorder. The data reported from each region were directly standardized according to European standards. Statistical significance was considered at a *p*-level of <0.05.

## Results

### Sample characteristics

Seven hundred and nine respondents (5.8%) reported frequent excessive sleepiness (>3 times per week), while 10.8% reported occasional sleepiness (1–2 times per week), and 2,200 reported rare sleepiness (18.6%). Tables 1–4 show the sample characteristics. Women in the study were more likely to report frequent sleepiness during the day compared to men. Social factors like marital status, education level, and employment status did not significantly impact sleepiness levels in the study.

**Table 1:** Social and demographic characteristics in the total sample and based on frequency of sleepiness.

Parameter	Total, *n* (%) or median (25–75 percentile)	Sleepiness <3 times/ week, *n* (%) or median (25–75 percentile)	Sleepiness ≥3 times/ week, *n* (%) or median (25–75 percentile)	*p*-level
Gender				
Men	5,141 (38,8)	4,880 (39,1)	261 (5,1)	*χ*^2^ = 100,6 *p* = 0.002
Women	8,114 (61,2)	7,594 (60,9)	520 (6,4)	
Age, years	48 (36–56)	48 (36; 56)	47 (36; 56)	0.838
25–34 years	22,8 (22.1–23.5)	472 (22.6)	2,558 (22.9)	*χ*^2^ = 0.435 *p* = 0.933
35–44 years	20,1 (19.4–20.8)	431 (20.6)	2,233 (20.0)	
45–54 years	27,4 (19.4–20.8)	569 (27.2)	3,057 (27.4)	
55–64 years	29,7 (29.0–30.5)	620 (29.6)	3,315 (29.7)	
BMI, m/kg^2^	27,1(23,8-31,2)	27.1 (23.8; 31.2)	27.4 (23.7; 31.2)	0.486
Waist-to-hip ratio obesity	5,839 (44.0)	945 (45.2)	945 (45.2)	0.221
Marital status				
Alone	1948 (14,8)	1836 (14,8)	112 (14,5)	*χ*^2^ = 0,643 *p* = 0,886
Married	8,705 (66,1)	8,198 (66,2)	507 (65,4)	
Divorced	1,624 (12,3)	1,522 (12,3)	102 (13,2)	
Widow	888 (6,7)	834 (6,7)	54 (7,0)	
Education				
Basic	554 (4,2)	514 (4,1)	40 (5,1)	*χ*^2^ = 4,143 *p* = 0,126
Second	6,814 (51,4)	6,396 (51,3)	418 (53,5)	
Higher	5,877 (44,4)	5,554 (44,6)	323 (41,4)	
Housing				
Own house	2080 (15,7)	1917 (15,4)	163 (21,0)	*χ*^2^ = 25,979 *p* < 0,001
Own flat	10,514 (79,5)	9,951 (79,9)	563 (72,4)	
Own room	266 (2,0)	244 (2,0)	22 (2,8)	
Residential house	209 (1,6)	192 (1,5)	17 (2,2)	
Other	156 (1,2)	143 (1,1)	13 (1,7)	
Employment				
Worker	10,137 (76,5)	9,568 (76,7)	569 (72,9)	*χ*^2^ = 7,406 *p* = 1,116
Never worked	122 (0,9)	113 (0,9)	9 (1,2)	
Unemployed	944 (7,1)	873 (7,0)	71 (9,1)	
Retired	1764 (13,3)	1,652 (13,2)	112 (14,4)	
Invalid	285 (2,1)	266 (2,1)	19 (2,4)	

**Table 2:** Laboratory parameters, mood and disease characteristics of the respondents in the total sample and based on frequency of sleepiness.

Parameter	Total, *n* (%) or median (25–75 percentile)	Sleepiness <3 times/ week, *n* (%) or median (25–75 percentile)	Sleepiness ≥3 times/ week, *n* (%) or median (25–75 percentile)	*p*
Laboratory parameters				
Fasting glucose, mmol/l	5,07 (4,60-5,55)	5.07 (4.60; 5.55)	5.07 (4.60; 5.55)	0.894
Creatinine, mmol/l	67,1 (61,0-74,7)	67.30 (61.00; 74.90)	66.20 (60.30; 73.60)	<0.001
GFR, ml per minute	100,5(90,9-110,6)	100,4 (90,6–110,5)	99,9 (90,4–10,9)	*р* = 0,387
Uric acid, mmol/l	293 (240–360)	295.00 (240.00; 360.00)	290.00 (233.00; 350.00)	0.024
Cholesterol, mmol/l	5,29 (4.55–6.07)	5.28 (4.55; 6.07)	5.30 (4.54; 6.11)	0.604
LDL, mmol/l	3,29 (2.64–4.01)	3.30 (2.65; 4.00)	3.29 (2.63; 4.03)	0.570
HDL, mmol/l	1,36 (1.16–1.61)	1.36 (1.15; 1.61)	1.39 (1.18; 1.65)	<0.001
Triglycerides, mmol/l	1,17 (0.83–1.71)	1.18 (0.83; 1.72)	1.13 (0.82; 1.68)	0.012
Mood				
Anxiety				
Abnormal	18,4 (17.8–19.1)	1900 (17.0%)	541 (25.9%)	<0.001
Borderline	27,9 (27.1–28.7)	3,049 (27.3%)	644 (30.8%)	
Normal	53,7 (52.8–54.5)	6,208 (55.6%)	905 (43.3%)	
Depression				
Abnormal	9,3 (8.8–9.8)	1,026 (9.2%)	209 (10.0%)	<0.001
Borderline	17,1 (16.5–17.8)	1850 (16.6%)	424 (20.3%)	
Normal	73,5 (72.8–74.3)	8,286 (74.2%)	1,458 (69.7%)	
SBP, mm Hg	130 (119–143)	130 (119–143)	129 (117; 142)	0.009
DBP, mm Hg	81 (74–89)	81 (74–89)	80 (73; 89)	0.074
HR, beat per minute	73 (67–79)	73 (67–79)	72,0 (66–79)	0.032
Arthrosis	4,701 (35,5)	4,453 (35,7)	248 (31,8)	*χ*^2^ = 4,995 *p* = 0,025
Rheumatoid arthritis	799 (6,0)	739 (5,9)	60 (7,7)	*χ*^2^ = 4,011 *p* = 0,045
Chronic bronchitis	1901 (14,3)	1768 (14,2)	133 (17,0)	*χ*^2^ = 4,880 *p* = 0,027
Asthma	415 (3,1)	380 (3,0)	35 (4,5)	*χ*^2^ = 4,991 *p* = 0,029
Hypertension	6,194 (46,7)	5,804 (46,5)	390 (49,9)	*χ*^2^ = 3,420 *p* = 0,038
Diabetes mellitus	559 (4,2)	515 (4,1)	44 (5,6)	*χ*^2^ = 4,122 *p* = 0,042
Myocardial infarction	271 (2,0)	253 (2,0)	18 (2,3)	*χ*^2^ = 0,281 *p* = 0,596
Stroke	258 (1,9)	234 (1,9)	24 (3,1)	*χ*^2^ = 5,519 *p* = 0,019
Coronary artery disease	2,382 (18,0)	2,179 (17,5)	203 (26,0)	*χ*^2^ = 13,704 *p* < 0,001
Other heart diseases	1,078 (8,1)	984 (7,9)	94 (12,0)	*χ*^2^ = 16,921 *p* < 0,001
Arrhythmia	2,382 (18,0)	2,179 (17,5)	203 (26,0)	*χ*^2^ = 36,227 *p* < 0,001
Ulcer disease	1,655 (12,5)	1,528 (12,2)	127 (16,3)	*χ*^2^ = 10,825 *p* = 0,001
Liver, gallbladder, and gastrointestinal tract conditions	4,924 (37,1)	4,579 (36,7)	345 (44,2)	*χ*^2^ = 17,546 *p* < 0,001
Renal disease	2,440 (18,4)	2,246 (18,0)	194 (24,8)	*χ*^2^ = 22,858 *p* < 0,001
Thyroid disease	1,649 (12,4)	1,526 (12,2)	123 (15,7)	*χ*^2^ = 8,339 *p* = 0,004
Parkinson’s disease	34 (0,3)	33 (0,3)	1 (0,1)	*χ*^2^ = 0,535 *p* = 0,464
Organ transplant	109 (0,8)	99 (0,8)	10 (1,3)	*χ*^2^ = 2,135 *p* = 0,144
Cancer	396 (3,0)	364 (2,9)	32 (4,1)	*χ*^2^ = 3,526 *p* = 0,06

DBP, Diastolic blood pressure; GFR, Glomerular filtration rate; HDL, High-density lipoprotein; HR, Heart rate; LDL, Low-density lipoprotein; SBP, Systolic blood pressure.

**Table 3:** Lifestyle characteristics in the total sample and based on frequency of sleepiness.

Parameter	Total, *n* (%)	Sleepiness <3 times/ week, *n* (%)	Sleepiness ≥3 times/ week, *n* (%)	*p*
Smoking	2,791 (21,1)	2,613 (21,0)	178 (22,8)	*χ*^2^ = 1,475, *p* = 0,225
Alcohol abuse	432 (3,4)	401 (3,2)	31 (4,0)	*χ*^2^ = 1,327, *p* = 0,252
Activity moderate/high	4,161 (31,4)	3,903 (31,3)	258 (33,0)	*χ*^2^ = 1,040, *p* = 0,308
High fat intake	2,758 (79,2)	2,557 (20,5)	201 (25,7)	*χ*^2^ = 12,236, *p* < 0,001
High salt intake	6,896 (52,0)	5,269 (52,2)	1,081 (47,3)	*χ*^2^ = 2,701, *p* < 0,001
Low vegetable and fruit index	5,310 (40,1)	4,983 (39,9)	327 (41,1)	*χ*^2^ = 1,131, *p* = 0,292
High sugar intake	6,299 (48,3)	5,889 (48,0)	410 (53,1)	*χ*^2^ = 7,443, *p* = 0,006

**Table 4:** Sleep complaints in the total sample and based on frequency of sleepiness.

Parameter	Total, *n* (%) or median (25–75 percentile)	Sleepiness <3 times/ week, *n* (%) or median (25–75 percentile)	Sleepiness ≥3 times/ week, *n* (%) or median (25–75 percentile)	*p*
Sleep duration, h	7,0 (7,0-8,0)	7 (7–8)	7 (6–8)	*р* < 0,001
Snoring	6,266 (47,3)	5,820 (46,7)	446 (57,1)	*χ*^2^ = 32,19 *p* < 0,001
Sleep apnea	1,176 (8,9)	1,036 (8,3)	140 (17,9)	*χ*^2^ = 84,138 *p* < 0,001
Sleep latency difficulties ≥3 times/week	2030 (15,3)	1,691 (13.6)	339 (43,4)	*χ*^2^ = 504,9 *p* < 0,001
Sleep maintenance difficulties ≥ 3 times/week	1753 (11,9)	1,266 (10,1)	307 (39,3)	*χ*^2^ = 597,5 *p* < 0,001
Sleeping pills ≥3 times/week	2,954 (22,3)	2,632 (21,1)	322 (41,2)	*χ*^2^ = 172,0 *p* < 0,001

The groups were well-matched on various health parameters, including age and age groups, weight (BMI), obesity classification by waist-to-hip ratio, fasting glucose, kidney function (glomerular filtration rate, GFR), cholesterol levels (total and LDL), and DPB.

People who reported frequent sleepiness showed some interesting health patterns (Table 3):

- Lower levels of: creatinine, uric acid, and triglycerides
- Higher levels of: HDL, anxiety, and depression
- Dietary habits: more likely to consume a diet high in sugar, salt, and fat
- Medical conditions:
- Lower rates of: arthrosis
- Higher rates of: rheumatoid arthritis, chronic bronchitis, asthma, hypertension, diabetes, stroke, coronary artery disease, arrhythmia, other heart diseases, ulcers, and conditions affecting the liver, gallbladder, gastrointestinal tract, kidneys, and thyroid diseases.

Table 4 demonstrates that respondents reporting frequent excessive sleepiness exhibited: shorter average sleep duration compared to those without excessive sleepiness, increased frequency of complaints regarding snoring and sleep apnea, difficulties with sleep initiation and maintenance, greater use of sleep medications.

### Predictors of frequent sleepiness

A backward multivariate logistic regression analysis identified significant predictors of sleepiness frequency after adjusting for continuous covariates [*χ*^2^(168) = 1,226, *p* < 0.001] (Table 5). Sleep complaints emerged as strong predictors, including frequent insomnia with sleep initiation and maintenance difficulties. Additionally, frequent use of sleep medication, and reports of sleep apnea and snoring were associated with increased sleepiness. Social and demographic factors including age, female gender, higher education, and retirement status were also included in the model.

**Table 5:** Multivariate logistic regression analyses of predictors with the probability of frequent sleepiness.

Predictors	Unadjusted		Adjusted	
	COR; 95% CI	*p*	AOR; 95% CI	*p*
Education: higher	0.734; 0.594–1.002	0.052	0.707; 0.667–0.997	0.047
Employment: retired	0.986; 0.854–1.140	0.850	1.246; 1.048–1.480	0.013
Age, years	0.998; 0.994–1.002	0.351	1.009; 1.004–1.015	0.001
Systolic blood pressure, mm Hg	1.002; 1.000–1.005	0.071	1.006; 1.002–1.009	0.003
Heart rate, beat per minute	1.005; 1.000–1.010	0.069	1.007; 1.002–1.013	0.007
Anxiety: borderline	1.335; 1.165–1.528	<0.001	1.146; 0.994–1.322	0.060
Anxiety: abnormal	1.946; 1.718–2.206	<0.001	1.431; 1.250–1.639	<0.001
High salt intake: yes	1.204; 1.091–1.328	<0.001	1.181; 1.065–1.309	0.002
Myocardial infarction: yes	1.125; 0.788–1.606	0.517	1.501; 1.023–2.203	0.038
Arrhythmia: yes	1.613; 1.435–1.815	<0.001	1.315; 1.154–1.498	<0.001
Other heart diseases: yes	1.673; 1.423–1.966	<0.001	1.389; 1.169–1.649	<0.001
Renal disease: yes	1.444; 1.283–1.626	<0.001	1.233; 1.087–1.399	0.001
Hypertension: yes	1.132; 1.025–1.249	0.014	1.169; 1.014–1.347	0.031
High-density lipoprotein, mmol/l	0.713; 0.623–0.815	<0.001	0.699; 0.607–0.805	<0.001
Sleep onset difficulties: ≥3 times/week	3.270; 2.915–3.666	<0.001	1.957; 1.684–2.273	<0.001
Sleep maintenance difficulties: ≥3 times/week	3.486; 3.083–3.943	<0.001	1.852; 1.575–2.179	<0.001
Sleeping pills: yes	1.928; 1.732–2.147	<0.001	1.473; 1.313–1.652	<0.001
Sleep apnea: yes	2.197; 1.900–2.542	<0.001	1.490; 1.269–1.751	<0.001
Snoring: yes	1.417; 1.284–1.564	<0.001	1.295; 1.161–1.445	<0.001

Beyond these, the analysis revealed further significant predictors:

- Mental health: presence of abnormal anxiety levels
- Laboratory parameter: high-density lipoprotein
- Dietary factors: high salt intake
- Comorbidities: arrhythmia, hypertension, myocardial infarction, other heart diseases, and renal disease.

The analysis quantified the impact of specific factors:

- For every one-year increase in age, the odds of frequent sleepiness increased by 1.009
- A 1 mmHg increase in SBP was associated with a 1.006 increased likelihood of frequent sleepiness
- Similarly, each additional heartbeat per minute increased the likelihood of frequent sleepiness by 1.007
- A 1 mmol/L decrease in HDL, the odds of frequent sleepiness increase by a factor of 0.699

The model fitted data well since the Deviance chi-square statistic was 1.0, and 0.87 for Pearson statistic. The model accounted for 10.3% (Nagelkerke *R*^2^) of the variance in excessive sleepiness and accurately classified 84.5% of cases.

Following the stratification of the age variable into discrete age groups, the results demonstrated that individuals in the 44–54 and 55–64 age categories exhibited a statistically significant association between age and the odds of frequent sleepiness, with 1.25 and 1.33 times increase, respectively, when compared to individuals in the 25–34 age category.

Direct comparison of predictors between women and men revealed distinct patterns of association (Supplementary Tables S2, S3). While sleep-related factors emerged as significant predictors in both sexes, the magnitude of these associations was generally stronger in men, with the exception of snoring. Beyond sleep factors, marked differences in predictors of frequent sleepiness were observed between genders. Hypertension, a significant predictor in both genders, was not associated with frequent sleepiness in either sex in this analysis. Compared to men, women exhibited a higher prevalence of several risk factors including age, education, systolic blood pressure, high salt intake, smoking, anxiety, myocardial infarction, and diabetes mellitus. Conversely, elevated low-density lipoprotein and creatinine levels were associated with increased risk exclusively in men. The impact of specific comorbidities on frequent sleepiness varied by sex. Arrhythmia, other heart diseases, and renal disease were more strongly associated with frequent sleepiness in men compared to women. Although high-density lipoprotein cholesterol demonstrated a protective effect in both genders, this association was more pronounced in men.

## Discussion

Our study indicates that a large number of the respondents from a representative sample of the Russian population experience EDS, with 5.8% reporting frequent episodes (>3 times per week) and 10.8% experiencing it occasionally (1–2 times per week). These percentages combined indicate that 16.6% of the surveyed population experiences EDS at least occasionally. This prevalence aligns with findings from international studies (3–5). However, previous Russian studies seem to show higher EDS prevalence. A study in Chuvashia (*n* = 2,161) reported 39.2% EDS using the Epworth Sleepiness Scale (ESS) (11). An online survey of young adults (*n* = 5,161) from Moscow and St. Petersburg found similar results with 40.9% experiencing EDS based on the ESS (12). Interestingly, a study by Khokhrina et al. focusing on sleep-disordered breathing (SDB) in Northwest Russia found no significant link between SDB and daytime sleepiness/fatigue. This suggests EDS in this Russian population might not be strongly tied to SDB (13).

Our analysis identified sleep disorder complaints as the strongest risk factors for EDS. This aligns with prior research demonstrating connections between EDS and conditions like insomnia and sleep apnea (3–5). Symptoms of insomnia can disrupt sleep quality and shorten sleep duration, leading to daytime sleepiness. However we did not know about insomnia diagnosis in those participants. Repeated breathing interruptions during sleep (characteristic of sleep apnea) cause fragmented sleep and contribute to excessive daytime sleepiness. While often used to treat sleep disorders, medications like sleeping pills can have side effects like EDS (14). Some people may use antidepressants as sleep aids, which may explain part of the sleep medication effect on sleepiness observed in our study. One limitation of the present study is the assessment of EDS using a single-item question. This measure may not have adequately differentiated between sleepiness, asthenia, and fatigue, potentially affecting the accuracy of our findings. While this approach was chosen for practical reasons, it is acknowledged that more comprehensive assessments, such as the ESS, would provide a more precise evaluation of EDS in future research. It is important to note that while the ESS is a widely used self-report measure of sleepiness, its correlation with objective measures of sleepiness, such as the Multiple Sleep Latency Test, has been reported to be low in some studies (15). This highlights the complexity of assessing sleepiness and the need for multiple methods to comprehensively evaluate this construct.

The association between retirement and EDS found in our study may be attributed to various factors, such as changes in daily routines, reduced social engagement, poor sleep hygiene or irregular sleep schedules, but these questions were not included in the survey. Breslau et al. showed that those who were employed had higher levels of sleepiness than those who were unemployed (16).

Sleep disturbances, particularly insomnia, can contribute to mental disorders by impairing brain neuroplasticity and stress immune pathways. This can lead to a state of allostatic overload, which can favor the development of mental disorders such as mood/anxiety disorders (17). Theorell-Haglöw, Lindberg and Janson’s study found that the combination of anxiety and depression was highly related to both EDS and fatigue (18). Among undergraduate medical students, the presence of excessive daytime sleepiness and caffeine consumption were significant risk factors for high levels of psychological distress (19).

The analysis identified only 5 significant medical conditions among the 18 investigated: arrhythmia, hypertension, myocardial infarction, other heart diseases, and renal disease. This finding aligns with existing research, as arrhythmia can disrupt sleep patterns and lead to daytime sleepiness. Studies suggest that poor sleep quality is prevalent in patients with atrial fibrillation, affecting approximately half of them (reference on arrhythmia and sleep quality). The severity of symptoms and the European Heart Rhythm Association score appear to be linked to a higher prevalence of sleep problems (20).

Myocardial infarction can lead to chronic health issues like heart failure, which can manifest in symptoms like sleep apnea, fragmented sleep, or even pain that disrupts sleep quality. These sleep disturbances could then contribute to EDS. Certain medications used to treat heart disease or manage post-infarction complications might have side effects including daytime sleepiness. Some underlying conditions, like obesity or obstructive sleep apnea, might increase the risk of both myocardial infarction and EDS (21). The association between hypertension and sleepiness is complex and not fully established. Hypertensive individuals often exhibit higher daytime sleepiness scores, which could be due to decreased sleep efficiency as influenced by hypertension. Even if high blood pressure does not directly cause sleepiness, it can contribute to factors that disrupt sleep, leading to daytime sleepiness. Certain medications used to treat hypertension can have side effects like fatigue or sleep disturbances. Hypertension is a risk factor and very often is associated with sleep apnea, which leads to daytime sleepiness (22).

Other heart diseases could include congestive heart failure, heart valve disease, congenital heart defects, pericarditis and cardiomyopathy. All these diseases can significantly disrupt sleep due to symptoms like shortness of breath, fatigue, and fluid retention. These issues can make it difficult to fall asleep, stay asleep, and achieve restful sleep, leading to daytime sleepiness (23).

Similar to arrhythmia, sleep disorders are common in patients with chronic kidney disease, particularly restless legs syndrome. The underlying mechanisms for these sleep disturbances are multifactorial and involve factors like uremia, anemia, metabolic acidosis, and co-existing medical conditions (reference on sleep disorders in renal disease). Research by Walker et al. (24) even found EDS in two-thirds of patients undergoing hemodialysis. These findings highlight the importance of considering coexisting medical conditions, when evaluating patients for EDS.

Daytime sleepiness is associated with several cardiovascular risk factors and conditions, but its direct relationship with HDL levels is less clear, with some studies suggesting an indirect association through conditions like diabetes mellitus and metabolic syndrome (25). Dyslipidemia, which often involves abnormal HDL levels, was found to be significantly associated with daytime sleepiness (26).

This study identified significant associations between sleepiness and two cardiac indicators: SBP and HR. Interestingly, a previous study by Goldstein et al. (27) also found a link between high sleepiness scores and elevated casual and sleep SBP. Their research even suggests that individuals with high sleepiness scores are more likely to develop hypertension within 5 years. However, it’s important to note that some research suggests a stronger association between sleepiness and changes in Heart Rate Variability (HRV) compared to just HR itself (28). HRV refers to the variation in time intervals between heartbeats and is considered a marker of autonomic nervous system function.

Lifestyle factors can significantly influence the incidence of excessive daytime sleepiness. Sedentary behavior, characterized by prolonged periods of inactivity, is linked to higher levels of daytime sleepiness (29). However we did not find a significant association between physical activity and sleepiness in our study. Consumption of foods rich in trans fat, sugar, and calories has been associated with increased daytime sleepiness (30). Castro and colleagues showed that changing the diet to a very low-calorie ketogenic for 4 weeks in obese patients reduced sleepiness up to 2 points of the Epworth sleepiness scale (31). But our study found that among dietary habits only high salt intake increased sleepiness, which can be influenced by various dietary habits and health conditions. High salt intake can lead to increased thirst and frequent nighttime urination, potentially disrupting sleep (32).

Women generally reported a higher prevalence of sleep disturbances and poor sleep quality compared to men (33–35). This is influenced by biological and psychological factors such as hormonal makeup, higher risk of depression, and rearing demands. Our study showed that while there are some common predictors of frequent sleepiness across genders, there are also notable differences. Women seem to have a broader range of significant predictors, including lifestyle factors (smoking, salt intake) anxiety and metabolic factors (diabetes), which are not significant in men. Conversely, men show significant associations with certain biochemical markers (creatinine, LDL cholesterol) that are not significant in women. There is considerable overlap between anxiety, age and sleep symptoms especially in women, which may reflect postmenopausal increase in sleep problems (35). These gender differences in predictors of frequent sleepiness could have important implications for understanding and managing sleep disorders. However, it’s important to note that these are observational findings, and further research would be needed to establish causal relationships and develop targeted interventions based on these gender differences.

### Limitations of study

Our study has some limitations that should be acknowledged. Data age: It’s important to note that the data used in this study is relatively old, being collected approximately 10 years ago. This temporal gap may limit the applicability of our findings to current populations, as sleep patterns, lifestyle factors, and societal norms may have changed over the past decade. Measurement approach: This study relied on a question regarding the frequency of sleepiness (“How often...”) rather than a validated measure of severity. While similar to the current definition of EDS and a question from the Pittsburgh Sleep Quality Index (36), further validation of this approach is recommended, at least with the Epworth sleepiness scale. Study design: The cross-sectional design limits our ability to establish causal relationships between the identified risk factors and EDS. Longitudinal studies are needed to understand how these factors might influence each other over time. While our model demonstrated good fit and classification accuracy, the relatively low explained variance (10.3%) suggests that other factors not included in our analysis may play important roles in determining sleepiness frequency. Self-reported measures: The use of self-reported data for EDS and sleep variables introduces potential reporting biases. Individuals might under- or over-report their experiences. We also did not use specific questionnaires for other sleep disorders like insomnia and sleep apnea. Our reliance on self-reported health conditions, including sleep apnea based solely on complaints, could introduce inaccuracies in our findings. Future studies might benefit from incorporating objective measures like sleep studies for sleep apnea and other sleep disorders diagnosis. Incomplete sleep assessment: The survey lacked questions on daytime napping, pregnancy, menopause, shift work, sleep timing, light exposure, social jet lag, electronic device use, sleep-promoting/disrupting dietary factors like caffeine intake and calorie consumption as factors influencing sleep patterns and daytime sleepiness. We asked about the frequency of sleeping pills but did not collect information on the medications groups or dosages used by participants. Some people may use antidepressants as sleep aids, which may explain part of the sleep medication effect on sleepiness observed in our study. Future research recommendations: Further research should consider incorporating objective sleep assessments and collecting data on the missing factors mentioned above. This would provide a more comprehensive picture of sleep quality and its relationship to EDS. Additionally, given the age of our data, replication studies using more recent data would be valuable to confirm if the relationships we observed still hold true in the current context. These limitations, particularly the age of the data, should be considered when interpreting our results. Despite these constraints, our study provides valuable insights into the factors associated with EDS and can serve as a foundation for future research in this area.

## Conclusion

Our study identified a notable prevalence of EDS in a representative sample of the Russian population, aligning with findings from other international studies. However, the prevalence observed here appears lower compared to some previous reports within Russia. This discrepancy warrants further investigation into potential regional variations or methodological differences. The analysis revealed a complex interplay of factors contributing to EDS frequency. Sleep complaints, particularly insomnia and sleep apnea, emerged as strong predictors. Demographics, mental health status, dietary habits, and co-existing medical conditions also played a significant role. The varying influence of comorbidities and physiological parameters between genders highlights the need for sex-specific approaches in assessing and managing daytime sleepiness. Understanding this multifactorial nature of EDS is crucial for clinicians. By addressing the underlying causes specific to each patient, healthcare professionals can develop more comprehensive treatment strategies to manage EDS and promote better sleep health in their patients.

## Data Availability

The datasets presented in this article are not readily available because of Russian Federal Law of July 27, 2006 №152 “Concerning Personal Data” (updated to September 1, 2015), Article 12 “Сross-border data transmission”. Requests to access the datasets should be directed to Mikhail Bochkarev bochkarev_mv@almazovcentre.ru.
