# Supplementary material for: Predictors of sleepiness in a large-scale epidemiology study ESSE-RF
Source: Front Neurol. 2024 Sep 4;15:1431821. doi: 10.3389/fneur.2024.1431821 (PMC11408917; doi:10.3389/fneur.2024.1431821)
Supplement: Supplementary file 1 [file Table_1.DOCX]

**Supplementary table 1.** Multivariate logistic regression analyses of predictors with the probability of frequent sleepiness including age groups

| Predictors | Unadjusted | | Adjusted | |
| --- | --- | --- | --- | --- |
|  | COR; 95% CI | p | AOR; 95% CI | p |
| Education: Higher | 0.734; 0.594 - 1.002 | 0.052 | 0.707; 0.667 - 0.997 | 0.047 |
| Employment: Retired | 0.986; 0.854 – 1.140 | 0.850 | 1.246; 1.048 – 1.480 | 0.013 |
| Age, years | 0.998; 0.994 – 1.002 | 0.351 | 1.009; 1.004 – 1.015 | 0.001 |
| Systolic Blood Pressure, mm Hg | 1.002; 1.000 – 1.005 | 0.071 | 1.006; 1.002 – 1.009 | 0.003 |
| Heart Rate, beat per minute | 1.005; 1.000 – 1.010 | 0.069 | 1.007; 1.002 – 1.013 | 0.007 |
| Anxiety: borderline | 1.335; 1.165 – 1.528 | < 0.001 | 1.146; 0.994 – 1.322 | 0.060 |
| Anxiety: abnormal | 1.946; 1.718 – 2.206 | < 0.001 | 1.431; 1.250 – 1.639 | < 0.001 |
| High Salt Intake: yes | 1.204; 1.091 – 1.328 | < 0.001 | 1.181; 1.065 – 1.309 | 0.002 |
| Myocardial Infarction: yes | 1.125; 0.788 – 1.606 | 0.517 | 1.501; 1.023 – 2.203 | 0.038 |
| Arrhythmia: yes | 1.613; 1.435 – 1.815 | < 0.001 | 1.315; 1.154 – 1.498 | < 0.001 |
| Other Heart Diseases: yes | 1.673; 1.423 – 1.966 | < 0.001 | 1.389; 1.169 – 1.649 | < 0.001 |
| Renal Disease: yes | 1.444; 1.283 – 1.626 | < 0.001 | 1.233; 1.087 – 1.399 | 0.001 |
| Hypertension: yes | 1.132; 1.025 – 1.249 | 0.014 | 1.169; 1.014 – 1.347 | 0.031 |
| High-Density Lipoprotein, mmol/l | 0.713; 0.623 – 0.815 | < 0.001 | 0.699; 0.607 – 0.805 | < 0.001 |
| Sleep Onset Difficulties: >3 times/week | 3.270; 2.915 – 3.666 | < 0.001 | 1.957; 1.684 – 2.273 | < 0.001 |
| Sleep Maintenance Difficulties: >3 times/week | 3.486; 3.083 – 3.943 | < 0.001 | 1.852; 1.575 – 2.179 | < 0.001 |
| Sleeping Pills: yes | 1.928; 1.732 – 2.147 | < 0.001 | 1.473; 1.313 – 1.652 | < 0.001 |
| Sleep apnea: yes | 2.197; 1.900 – 2.542 | < 0.001 | 1.490; 1.269 – 1.751 | < 0.001 |
| Snoring: yes | 1.417; 1.284 – 1.564 | < 0.001 | 1.295; 1.161 – 1.445 | < 0.001 |
| Age Group 35-44 Years | 0.939; 0.807 – 1.093 | 0.418 | 1.051; 0.897 – 1.232 | 0.536 |
| Age Group 45-54 Years | 0.958; 0.832 – 1.103 | 0.548 | 1.251; 1.069 – 1.465 | 0.005 |
| Age Group 55-64 Years | 0.947; 0.825 – 1.088 | 0.443 | 1.332; 1.110 – 1.598 | 0.002 |

**Supplementary table 2.** Characteristics of the association of predictors with the probability of sleepiness frequent in women

| Predictors | Unadjusted | | Adjusted | |
| --- | --- | --- | --- | --- |
|  | COR; 95% CI | p | AOR; 95% CI | p |
| Education: second | 1.275; 0.954 – 1.704 | 0.101 | 1.421; 1.044 – 1.935 | 0.026 |
| Education: higher | 0.708; 0.528 - 0.950 | 0.022 | 0.657; 0.478 – 0.902 | 0.009 |
| Employment: retired | 1.121; 0.715 – 1.758 | 0.618 | 1.957; 1.202 – 3.190 | 0.007 |
| Smoking Status: yes | 1.473; 1.231 – 1.761 | < 0.001 | 1.292; 1.066 – 1.565 | 0.009 |
| Age, years | 1.000; 0.994 – 1.005 | 0.838 | 1.009; 1.001 – 1.016 | 0.023 |
| Systolic Blood Pressure, mm Hg | 1.004; 1.001 – 1.007 | 0.012 | 1.006; 1.002 – 1.010 | 0.002 |
| High Salt Intake: yes | 1.234; 1.093 – 1.394 | 0.001 | 1.180; 1.038 – 1.340 | 0.011 |
| Anxiety: borderline | 1.427; 1.220 – 1.669 | < 0.001 | 1.218; 1.033 – 1.436 | 0.019 |
| Anxiety: abnormal | 2.122; 1.824 – 2.469 | < 0.001 | 1.550; 1.317 – 1.824 | < 0.001 |
| Myocardial Infarction: yes | 1.224; 0.678 – 2.210 | 0.502 | 2.331; 1.231 – 4.411 | 0.009 |
| Arrythmia: yes | 1.574; 1.369 – 1.809 | < 0.001 | 1.301; 1.115 – 1.519 | 0.001 |
| Other Heart Diseases: yes | 1.577; 1.300 – 1.914 | < 0.001 | 1.395; 1.134 – 1.714 | 0.002 |
| Renal Disease: yes | 1.382; 1.203 – 1.589 | < 0.001 | 1.181; 1.018 – 1.370 | 0.028 |
| Diabetes Mellitus: yes | 1.393; 1.070 – 1.813 | 0.014 | 1.453; 1.078 – 1.958 | 0.014 |
| High-Density Lipoprotein, mmol/l | 0.734; 0.619 – 0.869 | < 0.001 | 0.724; 0.601 – 0.872 | 0.001 |
| Triglycerides, mmol/l | 1.111; 1.028 – 1.202 | 0.008 | 1.108; 1.012 – 1.213 | 0.026 |
| Sleep Onset Difficulties: >3 times/week | 3.219; 2.807 – 3.691 | < 0.001 | 1.949; 1.627 – 2.333 | < 0.001 |
| Sleep Maintenance Difficulties: >3 times/week | 3.323; 2.875 – 3.842 | < 0.001 | 1.862; 1.536 – 2.257 | < 0.001 |
| Sleeping Pills: yes | 1.846; 1.616 – 2.109 | < 0.001 | 1.386; 1.201 – 1.598 | < 0.001 |
| Snoring: yes | 1.456; 1.289 – 1.644 | < 0.001 | 1.339; 1.168 – 1.536 | < 0.001 |
| Sleep apnea: yes | 2.180; 1.813 – 2.622 | < 0.001 | 1.463; 1.191 – 1.797 | < 0.001 |

**Supplementary table 3.** Characteristics of the association of predictors with the probability of sleepiness frequent in men

| Predictors | Unadjusted | | Adjusted | |
| --- | --- | --- | --- | --- |
|  | COR; 95% CI | p | AOR; 95% CI | p |
| Employment: retired | 1.400; 0.975 – 2.010 | 0.068 | 1.856; 1.267 – 2.716 | 0.001 |
| Heart Rate, beat per minute | 1.010; 1.002 – 1.018 | 0.019 | 1.012; 1.004 – 1.021 | 0.005 |
| Arrhythmia: yes | 1.578; 1.261 – 1.974 | < 0.001 | 1.319; 1.035 – 1.680 | 0.025 |
| Other Heart Diseases: yes | 1.821; 1.358 – 2.440 | < 0.001 | 1.490; 1.092 – 2.036 | 0.012 |
| Renal Disease: yes | 1.453; 1.151 – 1.833 | 0.002 | 1.309; 1.021 – 1.677 | 0.033 |
| Cholesterol, mmol/l | 0.990; 0.920 – 1.065 | 0.784 | 1.345; 1.045 – 1.732 | 0.021 |
| Low-Density Lipoprotein, mmol/l | 0.970; 0.890 – 1.057 | 0.481 | 0.763; 0.591 – 0.985 | 0.038 |
| High-Density Lipoprotein, mmol/l | 0.802; 0.628 – 1.025 | 0.078 | 0.581; 0.419 – 0.805 | 0.001 |
| Triglycerides, mmol/l | 0.993; 0.930 – 1.061 | 0.842 | 0.877; 0.785 – 0.980 | 0.021 |
| Creatinine, mmol/l | 0.996; 0.993 – 0.998 | 0.001 | 0.996; 0.993 – 0.999 | 0.008 |
| Sleep Onset Difficulties: >3 times/week | 3.211; 2.601 – 3.963 | < 0.001 | 1.908; 1.454 – 2.504 | < 0.001 |
| Sleep Maintenance Difficulties: >3 times/week | 3.681; 2.907 – 4.665 | < 0.001 | 2.014; 1.480 – 2.740 | < 0.001 |
| Sleeping Pills: yes | 2.076; 1.726 – 2.494 | < 0.001 | 1.692; 1.392 – 2.056 | < 0.001 |
| Snoring: yes | 1.406; 1.185 – 1.669 | < 0.001 | 1.211; 1.009 – 1.455 | 0.040 |
| Sleep Apnea: yes | 2.281; 1.799 – 2.895 | < 0.001 | 1.665; 1.289 – 2.151 | < 0.001 |
